# In Silico and In Vitro Comparison of Seven Closed and Semi-Closed Leaflet Designs for Transcatheter Heart Valve Replacements

**DOI:** 10.3390/bioengineering12101044

**Published:** 2025-09-28

**Authors:** Alexander Breitenstein-Attach, Marvin Steitz, Jordi Modolell, Sugat Ratna Tuladhar, Boris Warnack, Peter Kramer, Frank Edelmann, Felix Berger, Boris Schmitt

**Affiliations:** 1 Deutsches Herzzentrum der Charité, Department of Congenital Heart Disease—Pediatric Cardiology, Augustenburger Platz 1, 13353 Berlin, Germany; 2 Charité—Universitätsmedizin Berlin, Corporate Member of Freie Universität Berlin and Humboldt-Universität zu Berlin, Charitéplatz 1, 10117 Berlin, Germany; 3 German Centre for Cardiovascular Research (DZHK), 10785 Berlin, Germany; 4 Department for Cardiothoracic, Transplantation and Vascular Surgery, Hannover Medical School, Carl-Neuberg-Straße 1, 30625 Hannover, Germany; 5 Warnack Medconsult, Untere Wenkenhofstrasse 5, 4125 Riehen, Switzerland

**Keywords:** congenital heart disease, transcatheter heart valve replacement, pulmonary valve, heart valve design, pinwheeling, in vitro testing

## Abstract

**Purpose:** Transcatheter heart valve replacements (TVR) are typically designed in a closed shape with initial leaflet coaptation. However, recent studies suggest a semi-closed geometry without a predefined coaptation zone, relying on diastolic pressure and clinical oversizing of 10–20 % for closure. This approach may minimize pinwheeling, a phenomenon linked to early valve degeneration. **Method:** Seven valve geometries were assessed: one closed design (G0) and six semi-closed variations (G1–G6). The semi-closed designs differed in free edge shape (linear, concave, convex) and opening degree, defined as the relative distance from the leaflet to the valve center in the unloaded state. The opening degree was systematically increased across G1–G6, with G6 exhibiting the highest value. 30 mm valves were fabricated using porcine pericardium and self-expanding nitinol stents. Performance was assessed in a pulse duplicator system, evaluating transvalvular pressure gradient (TPG), effective orifice area (EOA), regurgitation fraction (RF) and a novel pinwheeling index (PI) which was validated by finite element simulations. **Results:** Finite element simulations demonstrated that semi-closed geometries achieve valve closure at a diameter reduction of >5%. In vitro tests confirmed these findings with more homogeneous coaptation and reduced pinwheeling. With increased opening degree the RF reduced significantly (RF_G0_ = 18.54 ± 8.05%; RF_G6_ = 8.22 ± 1.27%; *p* < 0.0001), while valve opening remained comparable (*p* = 0.4519). **Conclusions:** A semi-closed leaflet geometry enhances valve closure, reducing regurgitation and pinwheeling while preserving effective opening. With clinical oversizing, a higher opening degree improves coaptation and may enhance durability by mitigating structural deterioration, ultimately improving the long-term performance and lifespan of transcatheter valve replacements.

## 1. Introduction

Over the last two decades, the use of transcatheter heart valve replacement (TVR) has progressively increased as an alternative to surgical valve replacement (SVR), with a projected global implantation volume exceeding 130,000 procedures in 2025 [1]. Recent studies predict > 200,000 TVR implantations globally in 2030 [1,2,3]. To date, TVR has been performed in over three million patients across more than 80 countries and is surpassing SVR in frequency [2].

The primary distinction between transcatheter and surgical valve replacement lies in the anchoring mechanism of TVR, which relies on a stent for fixation within the vessel. To ensure adequate anchoring, the nominal diameter of the TVR before implantation must exceed the vessel diameter, thereby maintaining radial force and preventing prosthesis migration after implantation. This diameter difference, referred to as oversizing, is specified individually for each commercial TVR in its Instructions for Use. For example, the Edwards SAPIEN 3 requires an oversizing between 4% and 22%, Medtronic’s CoreValve specifies a range of 10% to 26%, and the Venus P Valve mandates oversizing between 6% and 14% [4,5,6].

However, TVRs are commonly produced in a closed position with leaflets initially coapting [4,5,6,7]. Due to the reduction in valve diameter following implantation as a result of oversizing, an excess of leaflet tissue is unavoidable. This excess material can lead to an inhomogeneous valve closure, in which the leaflets become entangled—a phenomenon known as pinwheeling. According to ISO 5840, pinwheeling shall be minimized, as it accelerates leaflet degradation and may compromise long-term valve function [8]. In response to this issue, recent literature has proposed manufacturing TVRs in a semi-closed shape, in which leaflets do not initially coapt [9,10]. The rationale behind this approach is that leaflet coaptation would occur only under conditions of oversizing and hemodynamic loading, thereby promoting a more homogeneous closure with reduced pinwheeling.

To evaluate the effectiveness of this semi-closed design, this study investigates six variations in semi-closed TVR geometries with incrementally reduced leaflet tissue and assesses their hydrodynamic performance using a ViVitro Pulse Duplicator System. The results are compared to a conventional fully closed TVR geometry to determine the impact of the semi-closed approach on valve functionality.

The valve designs and geometrical parameterizations within this study are based on a recent study by Breitenstein et al. [11]. In this publication we reviewed existing mathematical approaches and developed two geometric models: A conventional closed design with initially coapting leaflets and a novel semi-closed design, in which closure is achieved through hemodynamic pressure and clinical oversizing. The semi-closed design demonstrated improved performance due to reduced leaflet tissue, which minimized pinwheeling, confirming recent studies about valve geometry [9,10,12,13,14]. However, despite its advantages, pinwheeling was still observed in the semi-closed model. The findings from the previous study suggest that a further reduction in leaflet material may improve valve closure behavior and further reduce pinwheeling. This is why six geometry variations with reduced tissue material are assessed within this study to gain a deeper and more comprehensive understanding in valve kinematics and causes of pinwheeling [11].

In pediatric cardiology, pulmonary valve replacement is required in approximately 70% of patients, underscoring the clinical importance of this structure [15]. Hence, the present study focuses on the right heart and pulmonary valve. Due to the close morphological relationship between the pulmonary and aortic valves [16,17], the transferability of the results regarding optimal valve geometry towards adult cardiology and acquired heart valve diseases which affect aortic valves is anticipated and will be further explored in subsequent studies [18].

## 2. Materials & Methods

### 2.1. Parametrical Valve Geometry

**Figure 1:**
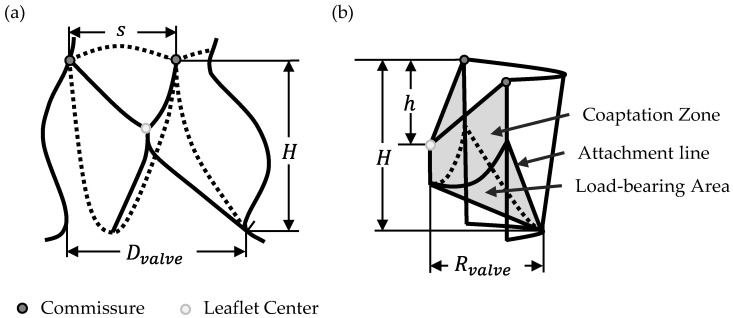
Geometrical description of the aortic valve [19,20,21]: (**a**) Aortic valve showing the side view of one leaflet with *D_valve_*: Valve diameter; *H*: Valve height; *s*: Commissural distance; (**b**) Schematic showing one leaflet in both open (transparent) and closed (grey) positions with *D_valve_*: Valve diameter; *H*: Valve height; *h*: Commissural height.

This study addresses the geometrical challenge of excess leaflet material, which contributes to pinwheeling and subsequent early degradation. To systematically modify valve geometry and mitigate this issue, two key geometrical parameters were defined to incrementally reduce leaflet tissue: the Opening Degree (*OD*), which quantifies the relative distance of the leaflet from the valve center, and the Free-Edge Shape, which can be either concave, linear, or convex. A detailed description of the derivation of the geometrical models for both the closed and semi-closed geometrical approach have been presented in our recent publication and serve as the fundamentals for the geometry variations within this study [11]. Figure 1 schematically illustrates the valve geometry along with fundamental geometrical parameters used to derive both *OD* and Free-Edge Shape, adapted from literature [19,20,21]. Figure 1a shows the valve in section view, while Figure 1b depicts a single leaflet in lateral view.

#### 2.1.1. Opening Degree

Following the parametric study of Xu et al., the parameter *OD* describes the relative retraction of the leaflet to the geometrical center point of the valve [12]. The parameter *OD* is calculated by the ratio of ∆*L*, which displays the distance from leaflet to valve center, and the valve radius *R_valve_*. It is mathematically defined in Equation (1).

(1) $$OD=\frac{∆L}{R_{valve}}*100\left[\%\right]=\frac{R_{valve}-L_{leaflet}}{R_{valve}}100\left[\%\right]\;$$

**Figure 2:**
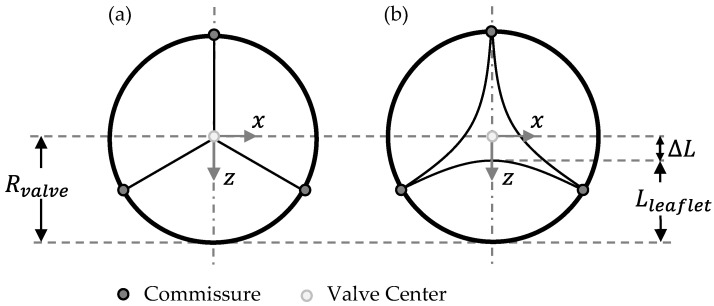
Modified leaflet parameterization for closed (**a**) and semi-closed (**b**) valve design based on Xu et al. [12].

All corresponding geometrical parameters are illustrated in Figure 2, which presents a two-dimensional top view of the closed (a) and semi-closed (b) valve designs. *R_valve_* represents the valve radius, *L_leaflet_* the maximum length of one leaflet and ∆*L* the length difference of these two parameters.

#### 2.1.2. Free-Edge Shape

**Figure 3:**
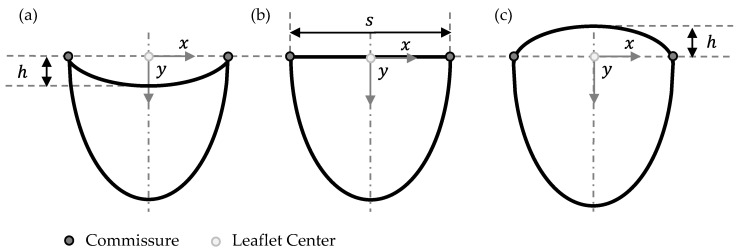
Free-Edge Shape variations in 2-dimensional front view of single leaflets for (**a**) concave, (**b**) linear and (**c**) convex leaflet shape.

The second geometry parameter to reduce leaflet material is the Free-Edge Shape, which is either concave, linear or convex, as shown in Figure 3a–c, respectively. It displays the outline of a single leaflet projected on a 2-dimensional plane in the frontal view.

Mathematically, the concave and convex free-edge curves are described in Equations (2) and (3), respectively. The linear free edge is in this coordinate arrangement equivalent to the *x*-axis and has no height value. Parameter *h* describes the vertical distance from the commissure to the leaflet center and *s* defines the horizontal distance between both commmissures of the leaflet (see Figure 1).

(2) $$y=-\frac{4*h}{s^{2}}*x^{2}+h$$

(3) $$y=\frac{4*h}{s^{2}}*x^{2}-h$$

Parameter *h* was defined as 10 % of the valve diameter which is equivalent to 3 mm. For *s*, $\sqrt{3}*R$ were applied, which is the edge length of a equilateral triangle with radius *R*. *R* refers to the valve radius.

#### 2.1.3. Geometry Variations

Within this study, the TVR with the closed geometry is designated as G0. The semi-closed geometries are labelled G1–G6, with an incremental reduction in leaflet material from G1 to G6. Quantitatively, this is illustrated in Table 1.

**Figure 4:**
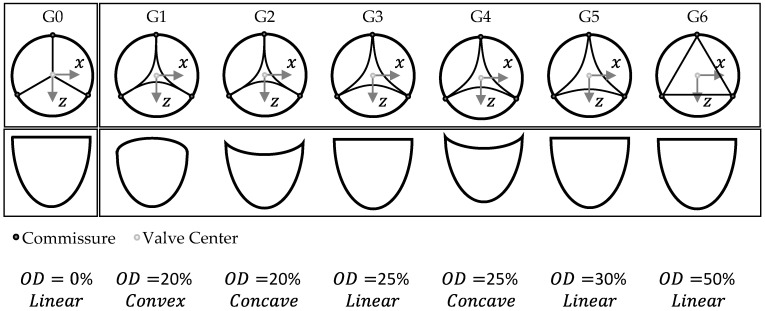
Graphical illustration of geometry variations G0–G6 in regard to their design parameters OD and Free-Edge Shape.

The geometrical variations are further illustrated in Figure 4.

### 2.2. TVR Fabrication

**Figure 5:**
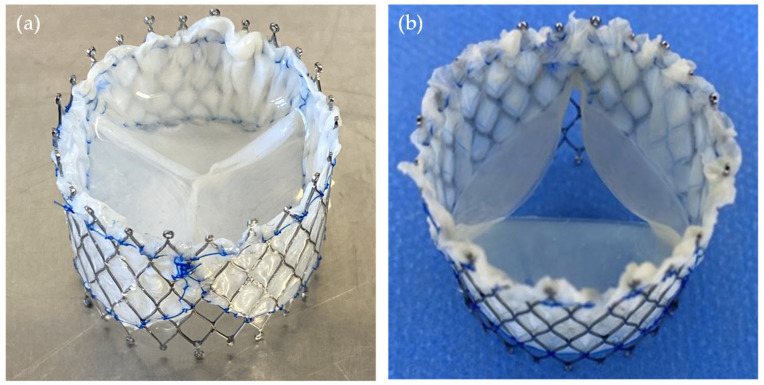
Manufactured prosthesis with (**a**) closed (G0) and (**b**) semi-closed (G5) design prior to implantation.

For each group G0–G6, three TVRs were fabricated to ensure a minimum statistical basis. Porcine pericardial tissue was used as leaflet material and re-shaped using a 3D-printed Mould. A nitinol stent with a diameter of 30 mm served as the structural support for all valve designs. The valves were crosslinked using a self-developed alternative to glutaraldehyde [22] which also relies on covalent collagen crosslinking. Figure 5 presents two exemplary valve prototypes, illustrating G0 geometry (Figure 5a) and G5 geometry variation (Figure 5b).

### 2.3. TVR Testing

#### 2.3.1. Test Conditions

As this research aims to enhance treatment options for patients with congenital heart disease—most of whom require a pulmonary heart valve replacement—the study was conducted under right heart conditions [23]. To assess valve performance, a commercially available pulse duplicator (ViVitro Labs Inc., Victoria, BC, Canada) was used for in vitro testing. It utilizes an electro-hydraulic piston pump unit that enables precise control of pulsatile flow and pressure waveforms to simulate physiological cardiac conditions. The programmable servo-driven mechanism allows adjustment of stroke volume, heart rate, and waveform shape, supporting reproducible assessment of valve function and leaflet dynamics under standardized conditions [24,25].

Physiological saline (0.9% sodium chloride in distilled water) was selected as test fluid due to its ability to provide reproducible mechanical and hydrodynamic conditions and reduced temperature sensitivity compared to common blood-mimicking fluids such as glycerol with xanthan gum [25,26,27]. The use of saline eliminates the risk of blood-related artifacts, simplifies experimental setup and increases comparability and standardization across laboratories, as saline is widely used for hydrodynamic assessments of heart valves [8,13,14,24,25]. For studies specifically focused on thrombogenicity or blood damage, blood analogs or actual blood may be required, but these are not necessary for short-term hydrodynamic testing [28,29]. This study solely focuses on mechanical parameters, such as regurgitant volume, effective area and pressure gradient, under controlled pulsatile flow conditions.

**Figure 6:**
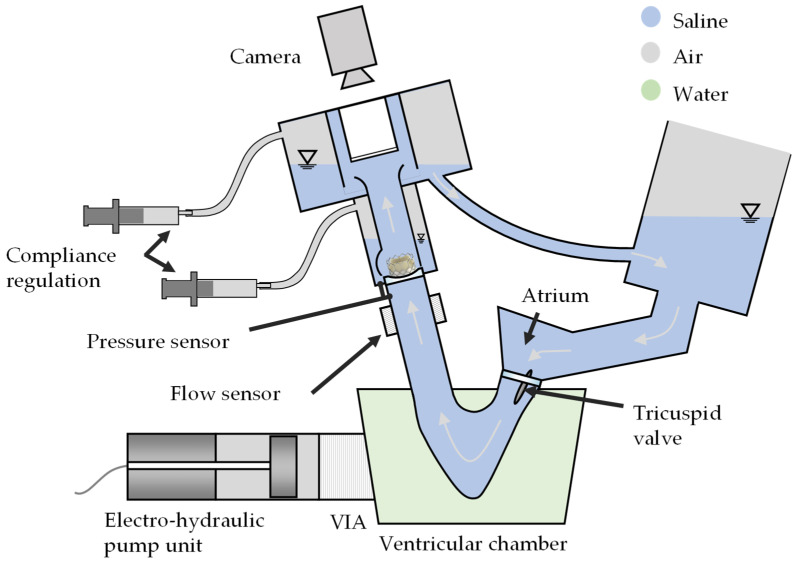
Schematic visualization of the ViVitro pulse duplicator.

To replicate the physiological pressure conditions of the right heart, modifications to the test bench were necessary, including the incorporation of a larger compliance volume. As a result of this adjustment, the heating element was removed, and the test fluid temperature was set to room temperature (23 ± 2 °C). A schematic representation of the pulse duplicator is shown in Figure 6.

Normotensive pulmonary pressure conditions were applied for testing, in accordance with ISO 5840. As specified in ISO 5840-1:2021, these conditions correspond to a right ventricle peak systolic pressure of 18–35 mmHg, a pulmonary artery end-diastolic pressure of 8–15 mmHg, and a peak differential pressure across the closed pulmonary valve of 13–28 mmHg [8]. Pathophysiological pressure conditions were not examined in this study.

The target conditions included a cardiac output of 5.0 L/min, a heart rate of 70 bpm, a mean arterial pressure (MAP) of 20 mmHg, and a systolic time span of 35%. These values represent medium physiological flow conditions, which are essential for evaluating both regurgitant volume and pressure difference, as outlined in ISO 5840-3:2021 [30].

The 30 mm TVRs were placed in a 26 mm annulus, resulting in clinically relevant 13.33% oversizing.

#### 2.3.2. Test Parameters

In order to comply with ISO 5840-3:2021, 10 consecutive cycles were captured for each valve. The following parameters were measured for each cycle [30]:

- Simulated cardiac output
- Cycle rate
- Systolic duration
- Forward flow volume
- Mean and RMS flow rates
- Mean pressure difference
- Effective orifice area
- Regurgitant volume, closing volume and leakage volume
- Mean arterial pressure over the whole cycle
- Appropriate qualitative photographic documentation

For evaluating the valve performance, the transvalvular mean pressure gradient (TPG) and effective orifice area (EOA) were used to describe the valve opening behavior, whereas the regurgitation fraction (RF) was assessed for the closing behavior. The TPG is the time-averaged arithmetic mean value of the pressure difference across a heart valve prosthesis during the positive differential pressure period of the cycle. As per ISO 5840-1:2021, the positive differential pressure period is the period when ventricular pressure is higher than the arterial [8]. The EOA is the valve’s ‘orifice area that has been derived from flow and pressure or velocity data [8] as per Equation (4) which is based on the Gorlin equation [31]:

(4) $$EOA=\frac{q_{v_{RMS}}}{51.6*\sqrt{\frac{TPG}{\rho }}}$$

ρ represents the fluid density (g/cm^3^), 51.6 displays an empirical derived constant and serves as a conversion factor from flow and pressure data to an opening area [31]. The fluid density is standardized to 1.005 g/cm^3^ [8]. $q_{v_{RMS}}$ is the root mean square forward flow (mL/s) during the positive differential pressure period, which is calculated with Equation (5):

(5) $$q_{v_{RMS}}=\sqrt{\frac{\int_{t_{1}}^{t_{2}}q_{v}{\left(t\right)}^{2}dt}{t_{2}-t_{1}}}$$

$q_{v}(t)$ is the instantaneous flow at time *t*, where *t*_1_ is time at start and *t*_2_ is time at end of positive differential pressure period, respectively.

To derive the RF, the ratio of regurgitant volume and the forward flow volume is calculated. The regurgitant volume is the sum of the closing and the leakage volume.

In order to evaluate the overall valve performance for each group, a mean value for each of the fluid dynamic testing parameters was calculated from all three valves of each group G0–G6. The resulting standard deviation displays the fluctuation within each group and test parameter. To qualitatively assess the valve performance, videographic recordings are taken from each TVR.

**Figure 7:**
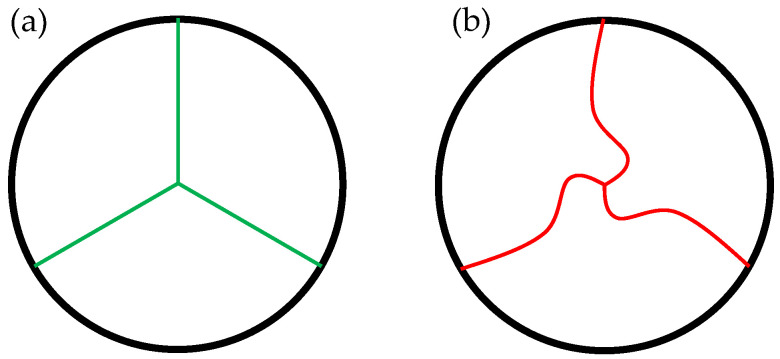
Valve top view (**a**) without and (**b**) with pinwheeling.

Another parameter to describe the geometrical valve closure is the so-called Pinwheeling Index (*PI*), introduced by Midha et al. [32] and visualized in Figure 7. It is defined by the ratio of the length difference of the actual ‘wheeled’ leaflet free edge length *L_actual_* (Figure 7b) versus the ideal length *L_ideal_* (Figure 7a) and the actual length.

Therefore, a high value corresponds to a high amount of pinwheeling. It is mathematically described in Equation (6).

(6) $$PI=\frac{L_{actual}-L_{ideal}}{L_{actual}}*100\;[\%]$$

Within this study, the TVR with the lowest and the highest amount of pinwheeling qualitatively derived from recordings were compared by post-processing the images and approximating the leaflet free edge utilizing the CAD Software Inventor Professional 2024 (Autodesk Inc., San Francisco, CA, USA).

#### 2.3.3. In Silico Analysis

To compare the approximated Pinwheeling Index derived from videographic recordings to a theoretical calculation, the *PI* was also obtained through an in silico verification using ANSYS Mechanical 2022 R2 (Ansys, Inc., Canonsburg, PA, USA). The model was discretized using 4-node full-integration shell elements for both the closed and semi-closed design G0 and G6, respectively. These elements were modeled as a homogeneous elastic material with an isotropic non-linear Young’s modulus, based on tensile tests conducted by Steitz et al. [22]. The Poisson’s ratio was set to 0.49 [33], and the density was taken as 1410 kg/m^3^ [34].

Since porcine pericardium was used for in vitro testing, a similar shell thickness of 200 µm was applied, as described by Labrosse et al. [35]. The valve diameter was reduced within the model by implementing nodal radial displacement, corresponding to an oversizing of 10% using a cylindrical coordinate system with the origin in valve center as displayed in Figure 2.

As this is a pure mechanical model to assess leaflet deformation and resulting pinwheeling, a physiological diastolic differential pressure of 20 mmHg according to ISO 5840 was applied [8]. Furthermore, the *PI* is the ratio between the ideal and actual coaptation line (see Equation (6)). Therefore, it is inherently scale-independent and a proportional downscale factor of 0.5 was applied for numerical simulation.

## 3. Results

### 3.1. Hydrodynamic Testing

Table 2 summarizes the measured test parameters along with the corresponding standard deviation between the three prostheses within each group.

**Figure 8:**
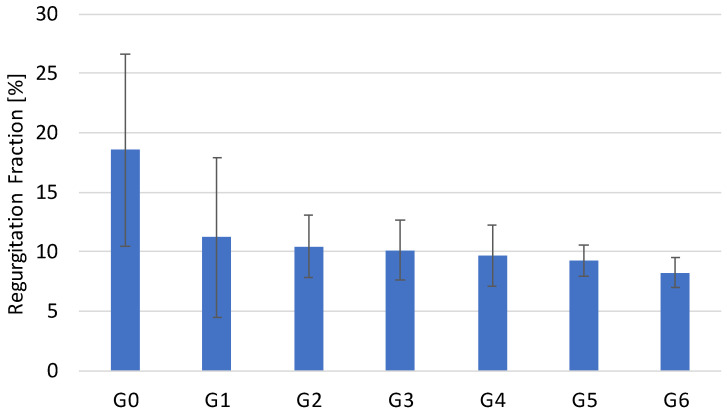
Graphical illustration of resulting Regurgitation Fraction for G0–G6.

**Figure 9:**
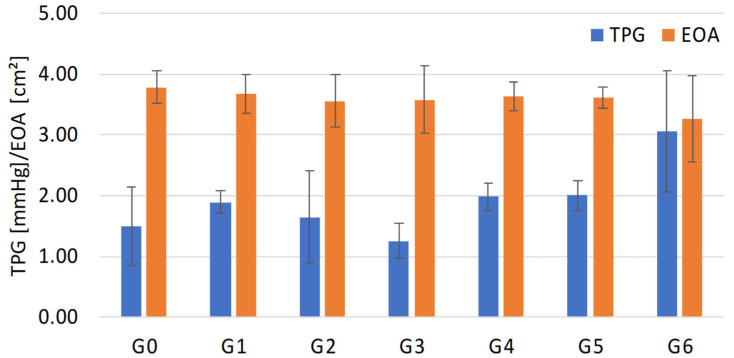
Graphical illustration of resulting TPG and EOA for G0–G6.

Figure 8 visualizes the results for the RF, whereas Figure 9 illustrates the results for TPG and EOA.

No significant difference was observed in valve opening performance across the tested geometries (ANOVA, *p* = 0.4519). In contrast, the regurgitant fraction (RF) exhibited a monotonic and statistically significant decrease with each reduction in leaflet tissue (ANOVA, *p* < 0.0001).

**Figure 10:**
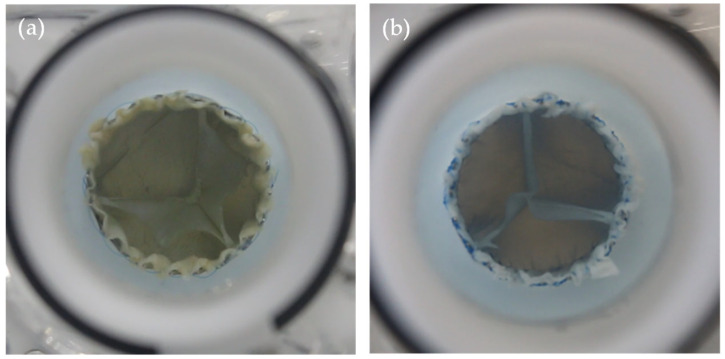
Top view of (**a**) G0 and (**b**) G6 during valve coaptation in pulse duplicator system.

The videographic recordings support the quantitative findings, demonstrating that G0 exhibits inhomogeneous leaflet closure with pronounced pinwheeling, whereas G6 achieves homogeneous coaptation with minimized pinwheeling. This observation is visualized in Figure 10. Full videos are available in the Supplementary Materials.

Based on the qualitative analysis of the videographic recordings, a *PI* of 13.63% was calculated for G0, whereas G6 exhibited a reduced *PI* of 10.21%.

#### In Silico Testing

The hydrodynamic results are quantitatively supported by numerical in silico simulations.

**Figure 11:**
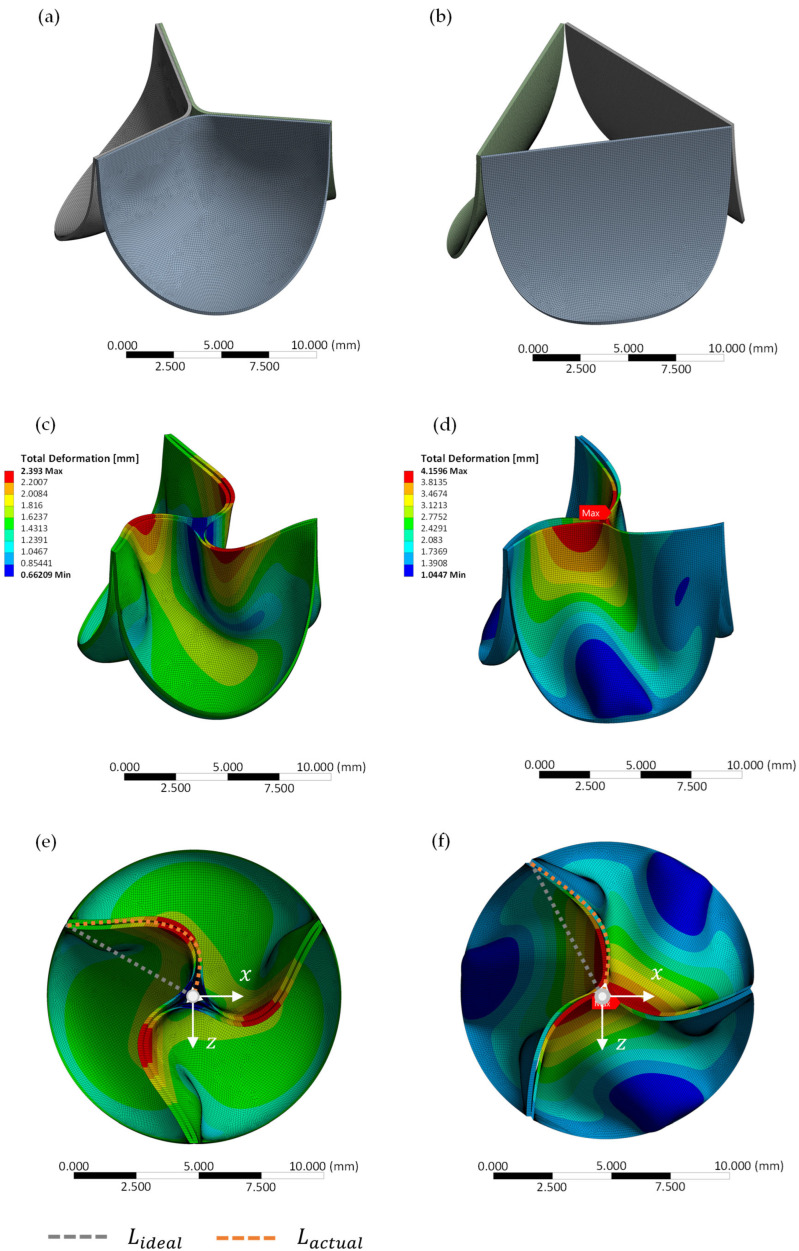
In silico comparison of G0 and G6 before crimping (**a**,**b**) and after applying oversizing from side view (**c**,**d**) as well as top view (**e**,**f**) with ideal free edge length *L_ideal_* (grey) and actual *L_actual_* (orange) for G0 and G6, respectively.

The simulations yielded a Pinwheeling Index of 10.84% for the closed geometry G0 and 7.1% for G6. Figure 11 visually illustrates this difference, showing the valve geometries at 0% oversizing before crimping (a,b) and after applying 10% oversizing, presented in both side view (c,d) and top view (e,f) for G0 and G6, respectively.

## 4. Discussion

Generally, the fabricated control group with closed geometry G0 was able to reproduce the unfavorable valve closure of current commercial TVR, such as Medtronic’s CoreValve or Edward’s SAPIEN 3 [7,36] which exhibit a high degree of pinwheeling and an increased RF. For each semi-closed geometry variation (G1–G6), the RF was significantly lower compared to G0. Furthermore, with progressive leaflet material reduction, the RF decreased as well, resulting in a monotonic decline across the different groups (G0–G6). This supports the hypothesis of Travaglino et al. that a semi-closed leaflet geometry is beneficial for valve closure and reduces pinwheeling [10].

The recorded videos support this explanation. In the closed configuration, the leaflets not only twist into one another, resulting in pinwheeling, but also close beneath the actual free-edge line, as shown in Figure 10a. As a result, surplus tissue remains above the effective coaptation line, which does not contribute to valve closure. This excess material prevented proper leaflet coaptation and reduced overall valve competence. Such variability likely explains the significant differences in standard deviation (F test, *p* < 0.0001): in some cardiac cycles, the valve achieved sufficient closure, whereas in others, the additional tissue obstructs neighboring leaflets and induces regurgitation. This finding confirms our previous research in regard to the closed valve design as well as Ma et al. who reported unfavorable stress distribution for convex free edge contours [11,37].

Despite this improvement, pinwheeling was still evident in the geometry variation with the least amount of leaflet tissue (G6). Therefore, further material reduction may be beneficial for optimizing valve closure. However, as the leaflet free edges in G6 form a triangular shape, the geometrical parameter OD cannot be increased beyond 50%. A potential modification could involve changing the Free-Edge Shape to a concave profile. Future studies should explore reduced oversizing and additional geometric adjustments, such as increasing the concave curvature radius or reducing leaflet height, to further minimize pinwheeling [12].

While RF exhibited a consistent decline with reduced leaflet material, no clear trend was observed for valve opening behavior, as transvalvular pressure gradient (TPG) and effective orifice area (EOA) fluctuated not significantly. The expected improvement in valve opening behavior due to reduced energy loss, as described by Kouhi and Morsi, could not be confirmed [9].

Beyond the clinically relevant parameters TPG, EOA, and RF, the novel parameter Pinwheeling Index, proposed by Midha et al. [32], was assessed to further characterize leaflet kinematics. In addition to the qualitative evaluation of valve function, as required by ISO 5840-1:2021, *PI* was introduced to quantify pinwheeling and provide a metric for assessing leaflet kinematics [32]. As expected from recent literature, the *PI* of the closed valve design G0 was 33.5% higher compared to the semi-closed design G6 during in vitro testing [9,10,12]. This significant difference between leaflet kinematics and resulting pinwheeling was further validated by in silico finite element analyses, where a relative reduction of 52.7 % in *PI* was observed between G0 and G6.

Possible explanations for the increased *PI* value in the in silico analysis include biological tissue variability (e.g., inhomogeneous thickness and fiber distribution) and simplifications in the material model used for simulation (e.g., isotropy and homogeneous thickness). These factors should be optimized in future studies to improve the accuracy of numerical predictions. To our knowledge, this is the first-ever combined in vitro and in silico investigation of *PI* in relation to geometrical heart valve design [32,38,39].

Another point to consider is that the present study was limited to short-term testing. Clinically relevant parameters TPG, EOA, and RF were recorded, and leaflet kinematics were additionally assessed using the pinwheeling index, in an attempt to provide a more informative prediction of long-term durability. Preventing stress concentrations in TVR is crucial, not only because they are known to accelerate structural valve deterioration, but also because they are associated with an increased risk of calcification in heart valves [40,41,42]. While it is reasonable to assume that valves performing better under short-term conditions are more likely to demonstrate superior long-term stability, this relationship is complex and multifactorial rather than strictly linear. Large clinical cohorts and multivariable analyses have shown that initial valve geometry that avoids prosthesis–patient mismatch and achieves low transvalvular gradients is associated with delayed onset and lower incidence of structural valve degeneration [43,44,45]. Accordingly, professional societies such as the Society of Thoracic Surgeons and the American College of Cardiology emphasize that high early gradients and suboptimal geometry are independent predictors of accelerated SVD and the need for reintervention [46,47].

At the same time, a recent computational and experimental study by Qiu et al. indicate that pinwheeling alone is a limited predictor of leaflet stress and strain distributions [48]. Although there is a direct link between pinwheeling and early valve degeneration, comparative analyses of transcatheter valve designs with varying coaptation heights and frame flexibility have shown that flexible frames may increase the pinwheeling index while reducing peak leaflet stress [48]. This dissociation suggests that pinwheeling and peak stress are influenced by different geometric and mechanical factors, and a high pinwheeling index does not necessarily correspond to elevated leaflet stress or accelerated degeneration. Therefore, pinwheeling should not be considered the sole surrogate for valve durability, but rather as one part of a comprehensive biomechanical assessment. [48,49].

Ultimately, the long-term functionality of transcatheter heart valves is determined by the interplay between leaflet kinematics, cyclic stress/strain distributions, and geometric deployment conditions. Incomplete stent expansion, frame distortion, or suboptimal sizing can increase both pinwheeling and localized stress concentrations, particularly at commissural tips and leaflet attachment points, thereby accelerating structural valve deterioration [48,50,51,52,53,54]. Conversely, design modifications that reduce peak stress may inadvertently increase pinwheeling. Achieving optimal valve durability therefore requires balancing minimal pinwheeling with low stress/strain levels, combined with precise deployment technique and geometric optimization. With respect to pinwheeling, the results of this study suggest that a semi-closed valve geometry may be advantageous. Regarding stress distribution, Bui et al. and Qiu et al. describe a concave free edge as particularly beneficial, while Visser et al. highlight the role of increased belly curvature and hinge length in enhancing long-term performance [38,48,55]. In terms of peak stress values and stress/strain distribution, a semi-closed valve design appears to perform better compared to the conventional closed geometry [9,10].

Taken together, these findings underscore that pinwheeling alone cannot predict valve durability. Nevertheless, it remains a meaningful design parameter and should be considered in conjunction with stress/strain distribution and deployment conditions. A comprehensive assessment integrating finite-element analysis, high-resolution strain mapping, and long-term experimental validation is essential to fully capture these relationships and guide the development of next-generation valve designs [48].

Future research should further explore the correlation between absolute *PI* values and corresponding valve degeneration considering also possible stress/strain distributions and device deployment. Establishing such a correlation could facilitate a severity classification of pinwheeling in relation to expected leaflet degradation. Ultimately, *PI* could become a clinically relevant parameter to characterize patient-prosthesis mismatch, aiding in personalized therapy strategies [56].

## 5. Limitations

This study has several limitations that should be considered when interpreting the results. One primary limitation is the use of biological tissue for valve fabrication. As pericardium is a natural material, there are inherent variations in fibre orientation and thickness between individual samples. These differences can lead to asymmetries in leaflet behavior, potentially affecting valve closure and pinwheeling. Although all valves were fabricated using tissue from the same anatomical region (above the left ventricle) to ensure consistency, no quantitative assessment of fiber distribution or thickness homogeneity was performed—only a visual inspection was carried out. Future studies should incorporate imaging-based thickness measurements or mechanical characterization of the leaflet material to quantify these variations prior to valve fabrication.

Another limitation derives from the test bench setup. As recommended by ISO 5840-3:2021, all TVRs were tested in a circular annulus to ensure standardized conditions [30]. However, the native anatomy of the pulmonary annulus is typically oval-shaped, meaning that the in vitro results may not fully translate to in vivo performance. The effect of non-circular geometries on valve function, including potential asymmetric leaflet coaptation and altered pinwheeling behavior, should be further investigated using anatomically more realistic mock vessels.

Additionally, all TVR prototypes were manufactured within a self-expanding nitinol stent, which was developed in-house. While the results demonstrate the feasibility of a semi-closed leaflet design for this type of stent, it remains unclear whether the same principles apply to balloon-expandable TVR. Unlike self-expanding stents, balloon-expandable valves rely on immediate vessel expansion rather than diameter reduction after implantation and continuous radial force application. Since oversizing requirements and mechanical behavior differ between the two stent types, future investigations should explore whether semi-closed geometries provide similar benefits for balloon-expandable devices.

Lastly, the finite element simulations used in this study introduce additional constraints. The material model assumed an isotropic and homogeneous tissue structure, neglecting the complex fiber orientation and anisotropic mechanical properties of pericardial tissue. Furthermore, leaflet thickness was kept constant across all simulations, whereas natural variations in thickness could influence the local stress distribution and leaflet deformation. The simulations also assumed a perfectly symmetric geometry, while in real-world applications, patient-specific variations in valve and vessel morphology lead to asymmetric loading and deformation. Future computational studies should integrate anisotropic material models, non-uniform thickness distributions and asymmetric boundary conditions approaches.

This study is limited by its exclusive focus on mechanical analysis of heart valve kinematics, without accounting for fluid mechanic effects or coupled fluid–structure interactions. The absence of FSI modeling means that critical fluid mechanical phenomena, such as vorticity, flow separation or other turbulence-inducing effects are not represented, which can significantly affect valve kinematics. The medical literature highlights that FSI is necessary to accurately simulate the dynamic behavior of both native and prosthetic heart valves, and its omission may limit the translational relevance and predictive accuracy of the results [57,58]. Nevertheless, it is not expected that fluid dynamic effects would mitigate existing pinwheeling. On the contrary, it is more likely that sinus vortices, which generally contribute to valve closure, would amplify this effect. For this reason, the fundamental statement on the resulting pinwheeling within this study remains valid, as it is expected to be exacerbated rather than improved. Therefore, the relative comparison between the valves under quasi-physiological mechanical loading can be considered accurate and should, in future studies, be extended to include fluid–structure coupling.

While these limitations highlight areas for refinement, they do not diminish the core findings of this study. Rather, they provide a roadmap for future research efforts aimed at further optimizing semi-closed leaflet geometries, improving in vitro test methodologies, and developing more physiologically accurate simulation models.

## 6. Conclusions

This study underscores the critical influence of oversizing on transcatheter heart valve performance, particularly due to its role in inducing pinwheeling and regurgitation. While closed leaflet designs remain the standard in commercial TVR, our results demonstrate that a semi-closed leaflet configuration, as proposed in recent literature, offers significant advantages in terms of valve closure dynamics. Across all in vitro tested geometries, systematic reduction in leaflet material led to a monotonic decrease in regurgitation fraction, confirming that a semi-closed design promotes more homogeneous coaptation with reduced leakage. Although leaflet reduction improved closure performance, no significant difference in valve opening behavior was observed.

Beyond conventional testing parameters such as transvalvular pressure gradient, effective orifice area, and regurgitation fraction, this study also quantitatively assessed leaflet kinematics using the Pinwheeling Index *PI* introduced by Midha et al. [32]. The results confirm that *PI* is significantly reduced in semi-closed geometries, reinforcing its potential as a quantitative marker for valve dynamics. Numerical in silico simulations further validated the observed geometric effects, supporting the applicability of *PI* as an additional performance indicator in TVR development. Understanding the correlation between pinwheeling and long-term valve durability could offer valuable insights into structural valve deterioration and failure mechanisms. A more detailed evaluation of *PI* in future studies may contribute to optimized TVR designs, ultimately reducing structural degeneration and improving prosthesis longevity.

These findings extend beyond TVR development and may influence heart valve prosthetic design, reconstruction, and neocuspidation. Optimizing leaflet geometry to minimize pinwheeling and patient-prosthesis mismatch could improve prosthetic durability, reduce re-operations, and enhance long-term clinical outcomes. Future research should focus on patient-specific adaptations of the semi-closed approach and refine computational models to further optimize valve performance under both physiological and pathophysiological conditions.

## Figures and Tables

**Table 1:** Geometry variations G0–G6 with corresponding parameter values for OD and Free-Edge Shape.

Geometry Variation	OD [%]	Free Edge Shape [-]
G0	0	Linear
G1	20	Convex
G2	20	Concave
G3	25	Linear
G4	25	Concave
G5	30	Linear
G6	50	Linear

**Table 2:** Mean values for each group G0–G6 and corresponding testing parameter RF, TPG and EOA.

	Hydrodynamic Parameter		
Geometry Variation	RF [%]	TPG [mmHg]	EOA [cm^2^]
G0	18.54 ± 8.05	1.49 ± 0.64	3.79 ± 0.26
G1	11.19 ± 6.75	1.89 ± 0.19	3.67 ± 0.33
G2	10.41 ± 2.62	1.64 ± 0.76	3.56 ± 0.44
G3	10.10 ± 2.51	1.25 ± 0.29	3.58 ± 0.55
G4	9.66 ± 2.55	1.98 ± 0.23	3.64 ± 0.23
G5	9.22 ± 1.28	2.00 ± 0.24	3.61 ± 0.17
G6	8.22 ± 1.27	3.06 ± 1.00	3.26 ± 0.71

Data were presented as mean ± standard deviation.

## Data Availability

All data on the assessed heart valve prostheses that support the findings of this study are included within this paper (see Table 2 for hydrodynamic test results per group and Hydrodynamic Testing along with In Silico Testing for quantitative pin-wheeling data). Raw data that support the findings of this study are available from the corresponding author, Alexander Breitenstein-Attach, upon reasonable request.

## Supplementary Materials

The following supporting information can be downloaded at: https://drive.google.com/drive/folders/1_iRVB0Vf2TKiJy_xDEfEpDpynBBrwKIw (accessed on 15 August 2025).

## Abbreviations

The following abbreviations are used in this manuscript:

EOA	Effective Orifice Area
ISO	International Organization for Standardization
OD	Opening Degree
PI	Pinwheeling Index
RF	Regurgitation Fraction
SVR	Surgical valve replacement
TPG	Transvalvular Pressure Gradient
TVR	Transcatheter valve replacement
